# Efficacy of Early Goal-Directed Therapy in Septic Shock Management: A Systematic Review

**DOI:** 10.7759/cureus.74358

**Published:** 2024-11-24

**Authors:** Prakash Acharya, Shikha Virani, Sumayya Afreen, Arvin Perthiani, Elizabeth Sangster, Nidhi Lanka, Iana Malasevskaia

**Affiliations:** 1 Internal Medicine, California Institute of Behavioral Neurosciences and Psychology, Fairfield, USA; 2 Medicine, Deccan College of Medical Sciences, Hyderabad, IND; 3 Obstetrics and Gynecology, California Institute of Behavioral Neurosciences and Psychology, Fairfield, USA; 4 General Surgery, Our Lady of Lourdes, Louth, IRL; 5 School of Medicine, St. George's University, St. George, GRD; 6 Psychiatry and Behavioral Sciences, California Institute of Behavioral Neurosciences and Psychology, Fairfield, USA; 7 Obstetrics and Gynecology, Private Clinic "Yana Alexandr", Sana'a, YEM; 8 Research and Development, California Institute of Behavioral Neurosciences and Psychology, Fairfield, USA

**Keywords:** early goal-directed therapy (egdt), mortality, randomized controlled trials (rcts), septic shock, survival outcomes, systematic review

## Abstract

Septic shock is a serious and life-threatening illness marked by potentially fatal and metabolic abnormalities, leading to high mortality rates in adult patients. Early goal-directed therapy (EGDT) was introduced as a systematic approach to manage septic shock through early, protocol-based hemodynamic optimization to improve outcomes. This systematic review aims to evaluate the efficacy of EGDT in managing adult patients with septic shock. A comprehensive search was conducted in multiple databases to identify relevant studies. Randomized controlled trials (RCTs), quasi-experimental, and observational studies with control groups were included. The quality of the studies was assessed using appropriate tools, and data was extracted for synthesis. This systemic review of 31 observational and RCT studies highlights the shortcomings of the one-size-fits-all EGDT strategy for septic shock. Although the preliminary research was encouraging, more recent studies have shown how important customized approaches are. Sophisticated monitoring methods such as tissue oxygenation and OER show potential in supporting customized hemodynamic therapy. Rigid protocols might not be the best option, but early intervention is essential. A move toward individualized treatment is necessary to enhance the prognosis of individuals suffering from septic shock. Future research should focus on well-designed RCTs, exploring comparative effectiveness, sustainability, and alternative monitoring techniques to refine the role of EGDT and develop more effective, evidence-based management strategies.

## Introduction and background

The management of septic shock and severe sepsis has been completely changed by early goal-directed therapy (EGDT), which introduced a systematic approach to hemodynamic improvement to enhance outcomes. EGDT is a protocol-based approach that targets particular physiological markers early during an illness. It was first proposed by Rivers et al. in 2001. To improve mortality rates, these measures include central venous pressure (CVP), mean arterial pressure (MAP), urine output, and central venous oxygen saturation (ScvO_2_) [1]. A significant improvement in the treatment of sepsis was made when there was a considerable decrease in mortality in patients undergoing EGDT as opposed to usual therapy [1]. The extensive implementation of EGDT and additional research into its effectiveness and suitability for a range of patient demographics were both sparked by this important study. The Surviving Sepsis Campaign guidelines, which were last revised in 2012, offered a global agreement on using EGDT as a cornerstone of sepsis treatment protocols [2]. To improve results and lower death rates, these guidelines stress how crucial it is to start EGDT as soon as severe sepsis or septic shock is identified. Despite early enthusiasm and guideline acceptance, research has been conducted to modify and validate the effectiveness of EGDT through comprehensive systematic reviews and meta-analyses. Recent systematic evaluations support the advantages of EGDT by demonstrating its correlation with higher survival rates and decreased organ dysfunction in sepsis patients [3]. Since the components of EGDT are now routine therapy, the mortality benefit has diminished, and more thorough RCTs are required to reevaluate its efficacy [4]. The simplified, goal-directed sepsis treatment protocol did not considerably reduce mortality, suggesting other factors may contribute to organ dysfunction in Zambian patients with severe sepsis [5]. The diversity in patient responses to EGDT methods was also highlighted with distinct risk factors linked to worse outcomes even after initial resuscitation attempts [6]. The purpose of this systematic review is to assess the available information on the effectiveness of EGDT in the treatment of adult patients with septic shock. This systematic review aims to evaluate the efficacy of EGDT in adult patients with septic shock, addressing the critical question: How effective is EGDT in improving survival rates compared to standard care? By synthesizing current evidence on the efficacy of EGDT, this review provides insights that can guide future research directions and inform clinical practice.

## Review

Method

Search Strategy

A systematic search was conducted between May 9, 2024, and May 25, 2024, to evaluate the efficacy of EGDT in managing septic shock in adult patients. The following databases were searched: PubMed, MEDLINE, Cochrane Library, Google Scholar, and Science Direct, and the following registers were searched: ClinicalTrials.gov and International Standard Randomized Controlled Trial Number (ISRCTN). A search strategy was developed using appropriate keywords, Boolean operators (AND, OR, NOT), and MeSH terms related to septic shock, EGDT, survival rates, and adult patients. To identify relevant studies, a systematic search was conducted following the Preferred Reporting Items for Systematic Reviews and Meta-Analyses (PRISMA) guidelines [7]. Keywords included "septic shock," "early goal-directed therapy," "survival rates," and "ICU admission” (Table 1). After removing duplicates using Zotero and organizing search terms, the initial search terms and combinations were available for reference in PubMed and other databases.

**Table 1:** Search strategy used for each database and register MEDLINE, medical literature analysis and retrieval system online; MeSH, medical subject headings; ISRCTN, International Standard Randomized Controlled Trial Number

Database	Search Strategy
Cochrane Library	adult OR MeSH descriptor: [Adult] explode all trees AND "early NEXT (goal-directed) NEXT(therapy)" OR "early NEXT (resuscitation)" OR "early NEXT (management)" AND MeSH descriptor: [Sepsis] explode all trees OR MeSH descriptor: [Shock, Septic] explode all trees OR "sepsis NEXT (syndrome)" OR "septic NEXT (shock)" OR sepsis AND MeSH descriptor: [Survival] explode all trees OR MeSH descriptor: [Survival Analysis] explode all trees OR MeSH descriptor: [Mortality] explode all trees OR "survival" OR "survivability" OR "mortality" OR "ICU" OR "death"
PubMed/Medline	("Adult"[MeSH Terms] OR "adult patient"[Text Word]) AND ("shock, septic"[MeSH Terms] OR "sepsis"[MeSH Terms] OR "sepsis syndrome"[Text Word] OR "SIRS"[Text Word] OR ("sepsis"[MeSH Terms] OR "sepsis"[All Fields])) AND ("early goal-directed therapy"[Text Word] OR "early resuscitation"[Text Word] OR "early management"[Text Word] OR "early hemodynamic optimization"[Text Word] OR "sepsis resuscitation"[Text Word]) AND (("Mortality"[MeSH Terms] OR "mortality rate"[Text Word] OR "death rate"[Text Word] OR "survival"[Text Word] OR "survival rate"[Text Word]) AND ("28-day"[Text Word] OR "28-day"[Text Word] OR "day 28"[Text Word]) AND "ICU admission"[Text Word]) OR "intensive care unit"[Text Word])
Google Scholar	"Early Therapy" AND "Septic Shock" AND "Adult Patients" "Goal Directed" -review, -meta-analysis, -pediatric
ScienceDirect	"Early Goal-Directed Therapy" AND "Septic Shock" AND "Efficacy"
ClinicalTrials.gov	Condition / Disease: Septic shock Intervention/Treatment: Early goal directed therapy
ISRCTN	Early Goal-Directed Therapy AND Septic Shock

Eligibility Criteria

Inclusion criteria: To be included, studies had to be randomized controlled trials (RCTs), quasi-experimental studies, or observational studies with a control group. The studies needed to be published in English, fully accessible as open access, and include adult patients diagnosed with septic shock. Additionally, the implementation of EGDT interventions and the reporting of 28-day or in-hospital mortality outcomes were required. To ensure methodological rigor, only studies with a "low" or "moderate" risk of bias were included, as determined by the Cochrane Risk of Bias tool for RCTs, the Newcastle-Ottawa Scale (NOS) (with a score of 70% or higher) for cohort studies, and the ROBINS-I tool for non-randomized studies.

Exclusion criteria: Studies were excluded if they involved non-adult patients, utilized interventions other than EGDT, or failed to adequately report survival outcomes within the specified timeframe. Additionally, studies with a high risk of bias or low methodological quality were excluded, along with case reports, animal studies, and studies without results, commentaries, editorials, and reviews. Studies were also excluded if the full text was not available or if they were assessed as having a "high" risk of bias, with an NOS score below 70%, or if the ROBINS-I tool indicated a high risk of bias.

Screening Process

Two independent reviewers (P.A. and E.S.) screened titles, abstracts, and keywords of all retrieved studies using a pre-defined selection criterion based on the inclusion/exclusion criteria established for the review. Disagreements between reviewers were resolved through discussion or by consulting a third reviewer (I.M.).

Final Selection

Following the screening and quality check process, 31 studies were included for detailed full-text review. Data on study design, patient characteristics, interventions, outcomes, and conclusions were extracted and synthesized.

Data Extraction and Quality Assessment

Both reviewers (P.A and E.S) independently extracted data using a predefined checklist and assessed methodological quality using tools based on the specific study design: NOS for non-randomized studies, like cohort studies evaluating aspects such as selection, comparability, exposure assessment, and outcome assessment. A study typically required a score of at least 70% on the NOS for inclusion in a systematic review [8]. Cochrane Risk of Bias Tool developed by the Cochrane Collaboration assessed bias in RCTs across domains like selection, performance, detection, attrition, and reporting bias, categorizing them as high, low, unclear, or no risk [9]. The Risk Of Bias In Non-randomized Interventions - of Studies (ROBINS-I) tool, similar to the Cochrane tool, evaluated bias in non-RCTs [10]. Studies with low and some concern of bias were included in the final analysis. The disagreements between reviewers regarding data extraction or quality assessment were resolved through discussion or by consulting a third reviewer (I.M.).

Results

**Figure 1:**
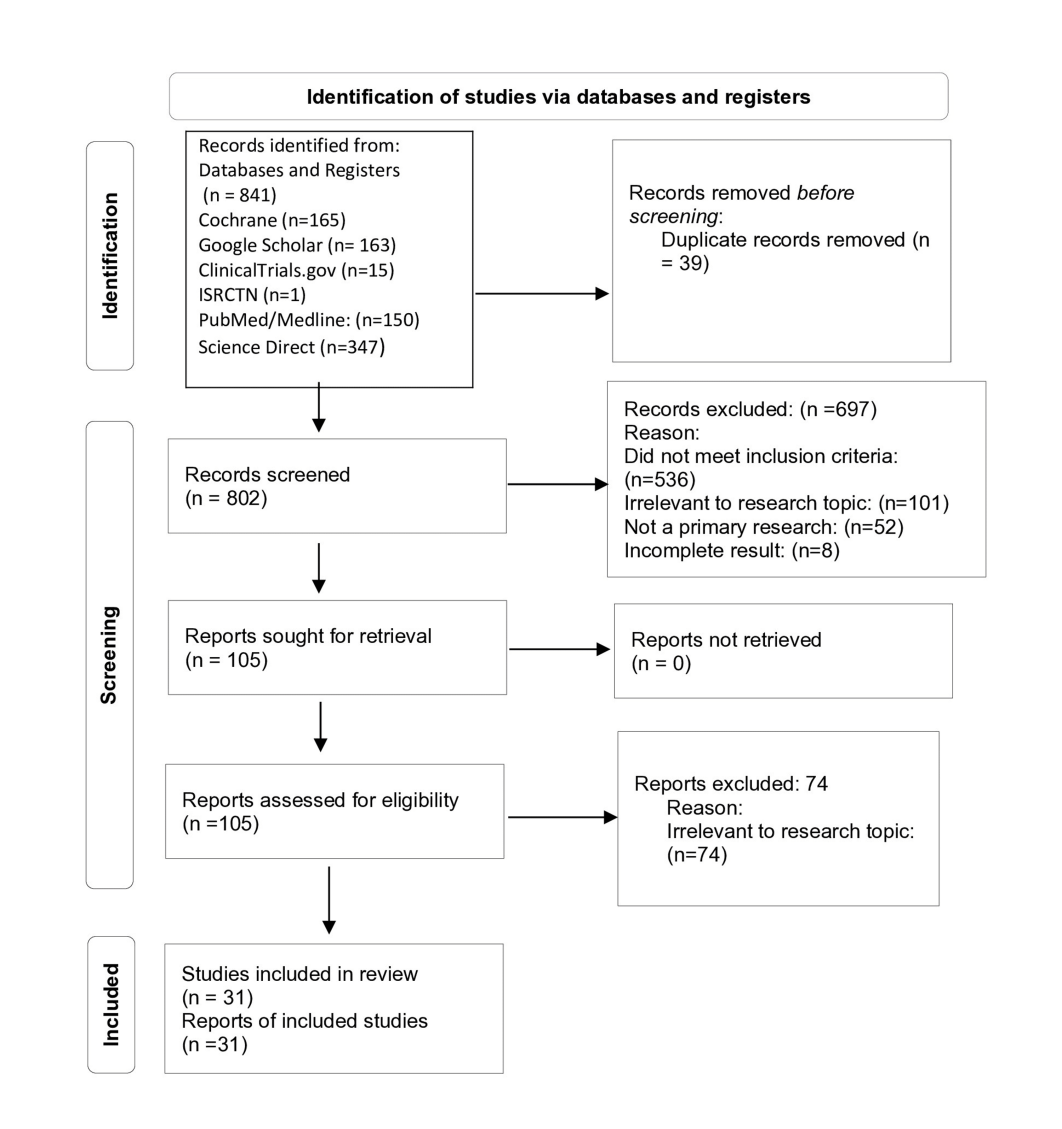
The PRISMA flow diagram PRISMA: Preferred Reporting Items for Systematic Reviews and Meta-Analyses [7].

Following the PRISMA guidelines, a total of 841 studies were identified through database and register searches, and 39 duplicate articles were removed using Zotero. The remaining 802 articles were then screened for inclusion or exclusion from the study. This was based on inclusion/exclusion criteria and relevance to the research aim by reviewing the abstract, method, result, and discussion. A total of 697 articles were subsequently removed due to not fulfilling the necessary requirements of the research aim or inclusion criteria. The remaining 105 articles underwent quality appraisal, of which 74 did not pass and were excluded from the study. Therefore, a total of 31 studies were potentially eligible for inclusion in this review. Figure 1 highlights these details.

Characteristics of Included Studies

The characteristics of the 31 studies included in this review are presented in Table 2. These studies employed a variety of designs, with 13 studies being experimental studies (RCTs) and 18 being observational studies. They focus primarily on severe sepsis and septic shock in diverse patient populations. The interventions and outcomes investigated varied across the studies, but the majority focused on the relationship between EGDT and survival rates at various time points.

**Table 2:** Characteristics of included studies EGDT, early goal-directed therapy; LOS, length of stay; PiCCO, pulse-indicated continuous cardiac output; OER, oxygen extraction ratio; ED, emergency department; RCT, randomized controlled trial; PAC, pulmonary artery catheter; CVC, central venous catheter; ScvO_2_, central venous oxygen saturation

Author(s)	Country	Date	Patient Group	Study Type	Outcome	Key Results	Comments
Rivers E, et al. [1]	USA	2001	Severe sepsis and septic shock	RCT	Mortality, morbidity	Significant benefits in outcomes for EGDT group	Pioneering study establishing EGDT efficacy
Colin et al. [11]	France	2012	Severe sepsis patients	Observational	28-day mortality prediction	Masseter and deltoid tissue oxygen saturation were strong predictors of 28-day mortality, unlike ScvO_2_	Masseter tissue oxygen saturation accurately identified patients with severe sepsis.
Yu et al. [12]	USA	1998	Patients 50-75 and >75 years	RCT	Survival rate	Improved survival in the 50-75 years group (p=0.01)	Over 75 years did not benefit from increased DO_2_, possibly overtreated.
ProCESS Investigators [13]	USA	2014	Patients with early septic shock	RCT	60-day mortality	No significant difference between groups (p=0.83)	Protocol-based EGDT, standard therapy, and usual care showed similar outcomes.
Higgins AM et al. [14]	Australia	2019	Patients with early septic shock	RCT	Quality of life and 1-year survival	EGDT compared with usual care did not reduce mortality nor improve health-related quality of life	Long-term follow-up shows no significant benefit of EGDT over usual care.
Gordon et al. [15]	USA	2012	Patients with septic shock	RCT	Cardiopulmonary effects	Vasopressin reduced heart rate but no change in cardiac output	Vasopressin and norepinephrine had similar effects on perfusion.
Coba et al. [16]	USA	2014	OHCA adults with bacteremia	Observational	Incidence of bacteremia, ED survival	38% incidence of bacteremia in OHCA adults, associated with severe metabolic derangements and high lactates. ED survival similar to severe sepsis	Further study needed to understand the cause-effect relationship between bacteremia and sudden cardiac arrest.
Mouncey et al. [17]	UK	2015	Patients with septic shock	RCT	90-day mortality, cost-effectiveness	No improvement in outcome with EGDT (p=0.90)	EGDT increased treatment intensity and costs without improving outcomes.
Protti et al. [18]	Italy	2018	Early sepsis patients	Observational	90-day mortality	Persistence of low Scvo_2_ associated with higher 90-day mortality due to underlying cardiac dysfunction	Subjects with Scvo_2_ <70% may benefit from tailored interventions aimed at normalizing systemic oxygen delivery.
Artero et al. [19]	Spain	2010	Community-acquired bloodstream infection with severe sepsis and septic shock	Observational	Prognostic factors of mortality	Hypoalbuminemia identified as a key prognostic factor along with severity of illness	Hypoalbuminemia should be considered in the prognostic assessment of these patients.
Jones et al. [20]	UK	2021	Emergency patients with sepsis	RCT	Feasibility of prehospital intervention	High recognition (92%) and randomization (74%) of eligible patients	Prehospital sepsis intervention by paramedics is feasible.
Mouncey et al. [21]	England	2015	Adults identified with early septic shock presenting to EDs	RCT	All-cause mortality at 90 days, cost-effectiveness	No significant difference in all-cause mortality at 90 days for EGDT compared with usual resuscitation. Costs were higher in EGDT group; probability of cost-effectiveness <30%	Highlights cost implications and effectiveness of EGDT in early septic shock management.
Lanspa et al. [22]	USA	2018	Critically ill patients presenting to ED with early septic shock	RCT	Early lactate clearance, substantial fluid administration	Patients already resuscitated before ICU arrival; future trials of echocardiogram-guided sepsis resuscitation may enroll in the ED	Emphasizes the feasibility and challenges of early fluid administration and lactate clearance.
Alhabashy et al. [23]	Egypt	2021	Patients with severe sepsis and septic shock	RCT	Mortality, ICU stay	Reduced mortality and ICU stay with echocardiography-guided management	Echocardiography-guided hemodynamic management showed improved outcomes.
Coskun et al. [24]	Turkey	2012	Patients with severe sepsis and septic shock	RCT	Hemodynamic parameters	No superiority of PAC over CVC for late period hemodynamic monitoring	PAC is costly, complex, and not recommended for routine use.
van Genderen et al. [25]	Netherlands	2015	Patients with septic shock	RCT	Hemodynamic parameters and perfusion	No significant difference in perfusion parameters between groups	Early peripheral perfusion-guided fluid therapy was not superior.
Xu et al. [26]	China	2014	Septic shock patients under controlled ventilation without arrhythmia	Observational	Volume responsiveness, fluid resuscitation, prognosis	SVV and PLR-ΔSV methods for evaluating volume responsiveness have similar effects on volume therapy and prognosis	Emphasizes importance of dynamic monitoring of volume responsiveness over specific evaluation methods.
Sweet et al. [27]	Canada	2010	Severe sepsis and septic shock patients in ED	Observational	ED sepsis protocol implementation	Improved care and outcomes for patients with severe sepsis and septic shock	Supports the implementation of sepsis protocols in ED for better management.
Trzeciak et al. [28]	USA	2008	Sepsis patients	Observational	Microcirculatory flow, organ failure	Increased microcirculatory flow during resuscitation associated with reduced organ failure at 24 hours	Suggests targeting microcirculation could improve outcomes in sepsis management.
Lu et al. [29]	China	2014	Septic shock patients	Observational	Disease severity, fluid resuscitation, mechanical ventilation, ICU stay	PiCCO-guided sepsis bundles reduced disease severity, lung water, and duration of mechanical ventilation and ICU stay	Highlights the clinical significance of modified sepsis bundles for improving patient outcomes.
Zhang et al. [30]	China	2012	Shock patients	Observational	Mortality	Fluid resuscitation according to EGDT-reduced mortality significantly	EGDT-based resuscitation strategy improves prognosis in shock patients.
Wawrzenia et al. [31]	Brazil	2015	Severe sepsis and septic shock patients in ICU	Observational	Mortality, LOS	Reduced mortality and LOS with EGDT in ICU setting	A simplified EGDT without ScvO_2_ is effective for sepsis management.
Lu Y. [32]	China	2013	Septic shock patients	Observational	Peripheral perfusion	Peripheral perfusion improved post-EGDT; not exactly reflected by systemic perfusion index	Indicates EGDT's effectiveness in improving tissue perfusion in septic shock patients.
Zhang et al. [33]	China	2016	Septic shock patients	Observational	Hepatic perfusion	No hepatic perfusion improvements after EGDT in early phase of septic shock	Suggests limited impact of EGDT on hepatic perfusion.
Na et al. [34]	Asia	2012	Sepsis patients in Asia	Observational	Mortality, ICU stay	Reduced mortality and ICU stay with early implementation of sepsis bundles	Timely adherence to sepsis bundles leads to better outcomes.
Zambon et al. [35]	Italy	2008	Severe sepsis and septic shock patients	Observational	Mortality, ICU stay	Correct application of the sepsis bundles was associated with reduced mortality and length of ICU stay	Emphasizes the importance of timely and correct implementation of sepsis bundles.
ARISE Investigators [36]	Australia	2014	Patients with early septic shock	RCT	90-day mortality	In critically ill patients presenting to the emergency department with early septic shock, EGDT did not reduce all-cause mortality at 90 days	Contrary to earlier studies, EGDT did not show mortality benefit in this cohort of early septic shock patients.
Cannon et al. [37]	USA	2009	Patients with severe sepsis and septic shock	Observational	In-hospital mortality, ICU stay, costs	Reduced in-hospital mortality and ICU stay with a multidisciplinary initiative	Institutional quality improvement initiatives significantly improve sepsis outcomes.
Park et al. [38]	Korea	2015	Severe sepsis and septic shock patients	Observational	Organ dysfunction, mortality	Initial low OER associated with severe organ dysfunction and high in-hospital mortality	OER should be considered in early-stage management for outcome prediction.
Hernandez et al. [39]	Chile	2005	Septic shock patients	Observational	Hemodynamic management	Norepinephrine-based algorithm effective for septic shock management with mortality rates comparable to other studies	Supports further trials comparing norepinephrine with dopamine.
Cabrera et al. [40]	USA	2015	Septic shock patients in ED	Observational	Patient outcomes	Protocol-based resuscitation did not improve outcomes	Calls for reconsideration of protocol-based approaches in septic shock management.

The primary outcomes studied were mortality rates, quality of life, and hemodynamic parameters in severe sepsis and septic shock patients. Initial findings from Rivers et al. showed significant mortality benefits from EGDT [1]. However, later studies, including ProCESS and ARISE, found no significant differences in mortality between EGDT and standard care, questioning the broad applicability of EGDT [13,36]. Key mortality predictors identified included masseter tissue oxygen saturation and hypoalbuminemia [11,19].

Additionally, the implementation of sepsis protocols was linked to improved outcomes [27,35]. Studies by Mouncey et al. and Gordon et al. highlighted that aggressive treatment strategies often did not lead to better outcomes, emphasizing the need for individualized approaches that balance efficacy and cost-effectiveness in sepsis management [15,21].

Risk of Bias Assessment

The risk of bias in the included studies (n=31) was assessed using appropriate tools for each study design (Table 3, Table 4). While some studies, particularly RCTs, exhibited concerns in specific domains (e.g., blinding and allocation concealment), the overall risk of bias was deemed acceptable for inclusion in the final review. Several RCTs, for instance, lacked adequate participant or researcher blinding, which could potentially inflate treatment effects. However, these studies were included despite these limitations due to the scarcity of high-quality evidence in this area.

**Table 3:** Cochrane risk of bias assessment for included RCTs D1, bias from the randomization process; D2: bias of deviations from intended interventions; D3, bias from missing outcome data; D4, bias from the measurement of the outcome; D5, bias in the selection of the reported result RCT, randomized controlled trial

Authors and Year	D1	D2	D3	D4	D5	Overall Risk of Bias
Rivers et al., 2001 [1]	Low risk	Low risk	Low risk	Low risk	Low risk	Low risk
Yu et al. [12]	Low risk	Some concerns	Low risk	Low risk	Some concerns	Some concerns
ProCESS Investigators, 2014 [13]	Low risk	Low risk	Low risk	Low risk	Low risk	Low risk
Higgins et al., 2019 [14]	Low risk	Some concerns	Low risk	Low risk	Low risk	Some concerns
Gordon et al., 2012 [15]	Low risk	Low risk	Low risk	Low risk	Low risk	Low risk
Mouncey et al., 2015 [17]	Low risk	Low risk	Low risk	Low risk	Low risk	Low risk
Jones et al., 2021 [20]	Low risk	Low risk	Low risk	Low risk	Low risk	Low risk
Mouncey et al., 2015 [21]	Low risk	Low risk	Low risk	Low risk	Low risk	Low risk
Lanspa et al., 2018 [22]	Low risk	Low risk	Low risk	Low risk	Low risk	Low risk
Alhabashy et al., 2021 [23]	Low risk	Low risk	Low risk	Low risk	Low risk	Low risk
Coskun et al., 2012 [24]	Low risk	Low risk	Low risk	Low risk	Low risk	Low risk
Van Genderen et al., 2014 [25]	Low risk	Low risk	Low risk	Low risk	Low risk	Low risk
ARISE Investigators, 2014 [36]	Low risk	Low risk	Low risk	Low risk	Low risk	Low risk

**Table 4:** NOS assessment for included observational studies NOS, Newcastle-Ottawa Scale

Authors and Year	Selection (Max 4)	Comparability (Max 2)	Outcome (Max 3)	Total Score (Max 9)
Colin et al., 2012 [11]	⭐⭐⭐	⭐⭐	⭐⭐⭐	⭐⭐⭐⭐⭐⭐⭐⭐
Coba et al., 2014 [16]	⭐⭐⭐	⭐	⭐⭐	⭐⭐⭐⭐⭐⭐
Protti et al., 2018 [18]	⭐⭐⭐	⭐⭐	⭐⭐⭐	⭐⭐⭐⭐⭐⭐⭐⭐
Artero et al., 2010 [19]	⭐⭐⭐	⭐⭐	⭐⭐⭐	⭐⭐⭐⭐⭐⭐⭐⭐
Xu et al. (2014) [26]	⭐⭐⭐	⭐⭐	⭐⭐⭐	⭐⭐⭐⭐⭐⭐⭐⭐
Sweet et al., 2010 [27]	⭐⭐⭐	⭐⭐	⭐⭐⭐	⭐⭐⭐⭐⭐⭐⭐⭐
Trzeciak et al., 2008 [28]	⭐⭐⭐	⭐⭐	⭐⭐⭐	⭐⭐⭐⭐⭐⭐⭐⭐
Lu et al., 2014 [29]	⭐⭐⭐	⭐⭐	⭐⭐⭐	⭐⭐⭐⭐⭐⭐⭐⭐
Zhang et al., 2012 [30]	⭐⭐⭐	⭐⭐	⭐⭐⭐	⭐⭐⭐⭐⭐⭐⭐⭐
Wawrzeniak et al., 2015 [31]	⭐⭐⭐	⭐⭐	⭐⭐⭐	⭐⭐⭐⭐⭐⭐⭐⭐
Lu et al., 2013 [32]	⭐⭐⭐	⭐⭐	⭐⭐⭐	⭐⭐⭐⭐⭐⭐⭐⭐
Zhang et al., 2016 [33]	⭐⭐⭐	⭐⭐	⭐⭐⭐	⭐⭐⭐⭐⭐⭐⭐⭐
Na et al., 2012 [34]	⭐⭐⭐	⭐⭐	⭐⭐	⭐⭐⭐⭐⭐⭐⭐
Zambon et al., 2008 [35]	⭐⭐⭐	⭐⭐	⭐⭐	⭐⭐⭐⭐⭐⭐⭐
Cannon et al., 2009 [37]	⭐⭐⭐	⭐⭐	⭐⭐	⭐⭐⭐⭐⭐⭐⭐
Park et al. (2015) [38]	⭐⭐⭐	⭐⭐	⭐⭐⭐	⭐⭐⭐⭐⭐⭐⭐⭐
Hernandez et al. (2005) [39]	⭐⭐⭐	⭐	⭐⭐	⭐⭐⭐⭐⭐⭐
Cabrera et al., 2015 [40]	⭐⭐⭐	⭐	⭐⭐	⭐⭐⭐⭐⭐⭐

Discussion

This systematic study found conflicting evidence about EGDT's effectiveness. One of the early trials, demonstrated a considerable decrease in mortality (from 46.5% to 30.5%), indicating that EGDT might be used as a standard of treatment [1]. Subsequent trials, however, have yielded inconsistent results about EGDT's efficacy. In patients presenting to the emergency department with early septic shock, EGDT compared with usual care did not lower mortality or enhance health-related quality of life at six or 12 months of follow-up [14].

Tissue-specific oxygenation measurements, specifically masseter and deltoid tissue oxygen saturation, were found to be highly predictive of 28-day mortality. In contrast, ScvO_2_ is less dependable [11]. This suggests that EGDT methods for early sepsis therapy should move toward localized monitoring strategies. Similar findings were made, which highlighted the necessity for tailored therapies by finding that sustained low ScvO_2_ during early sepsis was linked to greater 90-day mortality [18].

Numerous investigations looked into how sepsis outcomes were impacted by bacteremia and other illnesses. There is a significant rate of bacteremia in patients who experience out-of-hospital cardiac arrest (OHCA). This suggests that bacteremia and sudden cardiac arrest may be related, and it calls for more investigation into the early detection and management of sepsis [16]. Hypoalbuminemia was found to be a major prognostic factor in septic shock and severe sepsis, emphasizing the importance of taking it into account when developing patient management strategies [19].

There were no appreciable variations in the results of the comparison of techniques for assessing volume responsiveness in patients suffering from septic shock, indicating the superiority of dynamic monitoring over particular assessment techniques [26]. The therapeutic importance of customized sepsis bundles was highlighted by researchers who found that a modified surviving sepsis bundle treatment based on PiCCO decreased illness severity, lung water, and the length of mechanical ventilation and ICU stay [29]. Furthermore, evidence has been provided of the advantages of establishing a sepsis protocol in the ER, which enhances patient outcomes and care for those suffering from septic shock and severe sepsis [27].

The review also emphasizes the importance of microcirculatory flow and tissue perfusion. Targeting microcirculation may help improve organ function in sepsis, as it was found that higher microcirculatory flow during resuscitation was linked to decreased organ failure after 24 hours [28]. There are not any appreciable increases in hepatic perfusion following EGDT, which suggests that more research is necessary [33]. The effectiveness of EGDT in critical care settings was highlighted, which demonstrated decreased mortality and duration of stay using EGDT in ICU settings, even with a streamlined procedure that omitted ScvO_2_ [31].

The provided evidence of the efficacy of education and protocol adherence in the management of sepsis found that education and quality improvement initiatives increased sepsis bundle compliance, with team models outperforming non-team models [34]. The efficacy of a multidisciplinary sepsis quality improvement project was highlighted, which showed significant improvements in ICU stay and in-hospital mortality [37].

ProCESS, ProMISe, and ARISE are three pivotal trials that examined the effectiveness of EGDT and found no statistically significant changes in mortality between patients receiving protocol-based EGDT, protocol-based standard therapy, or usual care. While EGDT does not negatively impact outcomes, these trials found higher expenditures linked to it without a corresponding gain in patient outcomes [13,17,21,36]. This suggests that EGDT may not be cost-effective. The efficacy of echocardiography-guided management was demonstrated in studies, suggesting that tailored hemodynamic control may be more advantageous than rigid protocol-based techniques such as EGDT [22,23].

Park et al. emphasized the predictive value of the oxygen extraction ratio (OER), finding that, particularly in patients with normal ScvO2 levels but abnormal OER, an initial low OER correlates significantly with severe organ dysfunction and increased in-hospital mortality [38]. This emphasizes how important it is to use OER with ScvO_2_ as soon as possible in the management of sepsis in order to improve prognoses and inform treatment choices. Hernandez et al. provided evidence in favor of a norepinephrine-based algorithm for managing septic shock, showing mortality rates that were similar to those of alternative vasopressor regimens when illness severity was taken into account [39]. Their findings suggest future comparison trials with different medications and support the use of systematic vasopressor treatment to optimize hemodynamic stability in septic shock. Cabrera and Pinsky, on the other hand, discovered that, in emergency department settings, protocol-based resuscitation did not significantly improve outcomes when compared to flexible, personalized techniques [40]. This highlights the necessity for individualized, evidence-based solutions to effectively improve patient outcomes by highlighting the intricacy of standardizing techniques to accommodate the variety of clinical presentations of septic shock. Together, these findings highlight how crucial customized methods that combine thorough hemodynamic monitoring with flexible treatment plans are for improving sepsis care and lowering death rates.

Goal-directed therapy (GDT) significantly lowers overall mortality in sepsis patients, especially when started early, according to a meta-analysis by Gu et al. [41]. However, because of varying study quality, caution is advised. On the other hand, despite higher resource consumption and costs associated with EGDT, the PRISM Investigators discovered that EGDT did not yield superior outcomes compared to standard care across a heterogeneous patient and hospital sample [42]. Though the precise mechanisms underlying this advantage are yet unknown, Lu et al. did propose a mortality benefit with EGDT, particularly when treatments are given immediately during the first six hours [43]. Morton Hamer and Faught pointed out that increased mortality risks associated with EGDT in patients with severe sepsis may be explained by delayed administration of antibiotics rather than by the therapy itself [44].

Strengths and Limitations

This systematic review‘s strengths include a comprehensive search strategy, clear inclusion criteria, and diligent quality assessment. However, limitations include potential generalizability issues, the inclusion of observational studies, and possible language bias.

Future Research Directions

Future research should focus on conducting well-designed RCTs to establish causal relationships between specific EGDT protocols and survival outcomes in septic shock patients. Comparative effectiveness studies are also necessary to identify which components of EGDT offer the most significant benefits. Additionally, investigating the long-term impacts and sustainability of EGDT interventions is crucial for understanding their lasting effects on patient outcomes. Research should also explore the potential of alternative monitoring techniques, such as echocardiography, to enhance resuscitation strategies. Finally, examining the benefits of earlier intervention timing, particularly in pre-hospital or emergency department settings, could further improve the efficacy of EGDT in managing septic shock.

## Conclusions

The results of this systematic review pointed to a knowledge gap regarding the applicability of EGDT in managing septic shock. Although preliminary research indicated potential, more subsequent evaluations brought to light the drawbacks of a universal EGDT strategy. Future studies should concentrate on personalized medicine by investigating customized hemodynamic management, sophisticated monitoring methods (OER, tissue oxygenation, and microcirculation), and the possible advantages of early and efficient therapy. This shift toward more individualized care has the potential to improve outcomes for people suffering from septic shock.
